# Off-Stoichiometric Reactions at the Cell–Substrate Biomolecular Interface of Biomaterials: *In Situ* and *Ex Situ* Monitoring of Cell Proliferation, Differentiation, and Bone Tissue Formation

**DOI:** 10.3390/ijms20174080

**Published:** 2019-08-21

**Authors:** Giuseppe Pezzotti, Tetsuya Adachi, Francesco Boschetto, Wenliang Zhu, Matteo Zanocco, Elia Marin, B. Sonny Bal, Bryan J. McEntire

**Affiliations:** 1 Ceramic Physics Laboratory, Kyoto Institute of Technology, Sakyo-ku, Matsugasaki, Kyoto 606-8585, Japan; 2 Department of Orthopedic Surgery, Tokyo Medical University, 6-7-1 Nishi-Shinjuku, Shinjuku-ku, Tokyo 160-0023, Japan; 3 The Center for Advanced Medical Engineering and Informatics, Osaka University, 2-2 Yamadaoka, Suita, Osaka 565-0854, Japan; 4 Department of Immunology, Graduate School of Medical Science, Kyoto Prefectural University of Medicine, Kamigyo-ku, 465 Kajii-cho, Kyoto 602-8566, Japan; 5 Department of Dental Medicine, Graduate School of Medical Science, Kyoto Prefectural University of Medicine, Kamigyo-ku, Kyoto 602-8566, Japan; 6 SINTX Technologies Corporation, 1885 West 2100 South, Salt Lake City, UT 84119, USA

**Keywords:** silicon nitride bioceramic, alumina bioceramic, titanium alloy, X-ray photoelectron spectroscopy, *in situ* Raman spectroscopy

## Abstract

The availability of osteoinductive biomaterials has encouraged new therapies in bone regeneration and has potentially triggered paradigmatic shifts in the development of new implants in orthopedics and dentistry. Among several available synthetic biomaterials, bioceramics have gained attention for their ability to induce mesenchymal cell differentiation and successive bone formation when implanted in the human body. However, there is currently a lack of understanding regarding the fundamental biochemical mechanisms by which these materials can induce bone formation. Phenomenological studies of retrievals have clarified the final effect of bone formation, but have left the chemical interactions at the cell–material interface uncharted. Accordingly, the knowledge of the intrinsic material properties relevant for osteoblastogenesis and osteoinduction remains incomplete. Here, we systematically monitored *in vitro* the chemistry of mesenchymal cell metabolism and the ionic exchanges during osteoblastogenesis on selected substrates through conventional biological assays as well as via *in situ* and *ex situ* spectroscopic techniques. Accordingly, the chemical behavior of different bioceramic substrates during their interactions with mesenchymal cells could be unfolded and compared with that of biomedical titanium alloy. Our goal was to clarify the cascade of chemical equations behind the biological processes that govern osteoblastogenic effects on different biomaterial substrates.

## 1. Introduction 

It is well established that bioceramics could be bioactive in terms of both osteoblastogenesis and osteoinductivity [1]. However, to acquire conclusive evidences that osteoinductive bioceramics can provide a valid alternative to autologous bone and osteogenic growth factors, a complete understanding of the chemical mechanisms behind the interaction between cells and the bioceramic surface is needed. In this context, we notice that, within the field of biomaterials, it is common to classify oxide (e.g., alumina) and non-oxide ceramics (e.g., silicon nitride) as fully bioinert materials while only synthetic apatites and calcium phosphates are considered to be bioactive [1,2,3]. We shall instead provide clear evidence that those oxide and non-oxide ceramics are not bioinert. Conversely, they may be either supportive (bioactive) or detrimental to differentiation and metabolism of mesenchymal progenitor cells. After an initial proposal of osteoinductivity for calcium phosphate containing biomaterials [4,5], only one study has proposed osteoinductivity for alumina ceramics [6]; however, several studies have favored titanium as an osteoinductive substrate [7,8]. Recent *in vivo* and *in vitro* studies [9,10] have indicated that silicon nitride, a non-oxide bioceramic previously considered to be fully bioinert [1], is instead a formidable stimulator of osteoblastogenesis and osteoinductivity. The mechanisms of osteoinduction by the above biomaterials have been phenomenologically covered by the above publications, but the fundamental chemistry driving osteoblastogenesis and the successive bone formation needs additional elucidation. For more than 50 years bone biologists have embarked on efforts to understand the dynamic processes of differentiation and energetics of bone cells. However, the initial investigations of substrate utilization by bone cells were mainly focused on finely tuning the culture conditions for supporting collagen and mineral production [11]. Later, the focus shifted to hormonal regulation [12]. Currently, the search targets the role of substrates in anabolic treatments for osteoporosis and the enhancement of the work of the osteoblast through ionic alteration of osteoblast metabolism [13,14]. In this study, we re-examine and compare the surface chemistries of alumina, silicon nitride, and Ti6Al4V titanium alloy in this latter optics. Oxide and non-oxide bioceramics were selected for this investigation because they are presently used in joint arthroplasty and spine arthrodesis, respectively. Both bioceramics are considered as innovative choices with respect to titanium alloy, which is widely used in both the above applications. For this latter reason, we selected the Ti6Al4V alloy as the most appropriate substrate for comparative purpose.

The focus of this paper is on the ionic exchange at the interface between mesenchymal cells and different substrates. The aim of this study is to clarify which off-stoichiometric reactions take place at the biomolecular interface of bioceramics and how they differ between alumina (Al_2_O_3_) and silicon nitride (Si_3_N_4_) bioceramic substrates, demonstrating how the former stresses the cells in a similar way as titanium alloy, while the latter supports cell metabolism and bone formation.

## 2. Results

### 2.1. Substrate Surface Modifications in Aqueous Environment

**Figure 1:**
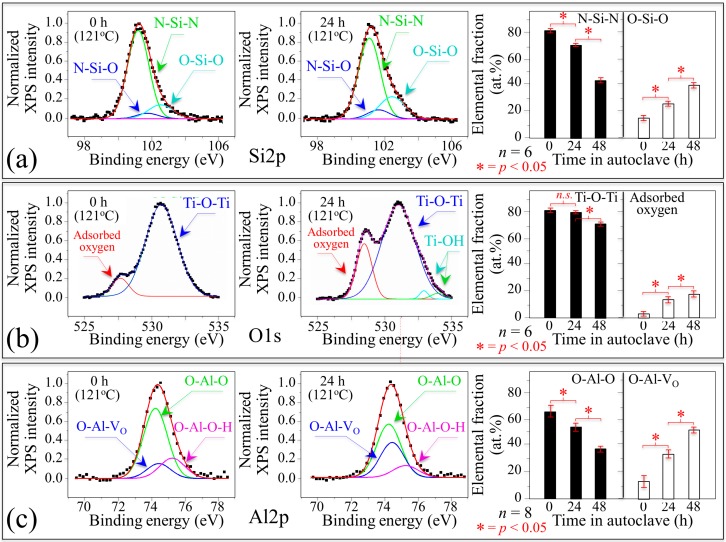
X-ray photoelectron spectroscopy (XPS) analyses of the investigated substrates before (left) and after (center) accelerated tests in water vapor environment, and plots summarizing elemental fraction results at different autoclaving times (right): (**a**) Si2p core spectrum of silicon nitride; (**b**) O1s core spectrum of Ti6Al4V alloy; and (**c**) Al2p core spectrum of alumina, with the related deconvolutions and quantitative time dependencies. Statistics were made according to the one-way Analysis of Variance (ANOVA) to assess the plots on the right side of each section.

The experiments described in this section challenge the notion that alumina oxide and silicon nitride non-oxide bioceramics remain completely inert in an aqueous environment. The substrate samples used in this study had surfaces with comparable average values of roughness: 0.32 ± 0.02, 0.10 ± 0.01, and 0.29 ± 0.04, for Si_3_N_4_, Al_2_O_3_, and Ti6Al4V alloy substrates, respectively. Figure 1a–c show the variations of X-ray photoelectron spectroscopy (XPS) Si2p core spectrum of silicon nitride, O1s core spectrum of Ti6Al4V alloy, and Al2p core spectrum of alumina with time in water vapor environment, respectively. The core spectra in the respective sections, which compare the as-received and 24 h-exposed surfaces, were deconvoluted into peak components representing the respective bonds, as shown by the labels of the figure [15,16,17,18,19,20]. The plots on the left side of each section give the trends with time of the population of individual bond components in terms of elemental fractions.

XPS data showed that all the investigated substrates underwent significant variations in their surface chemistry upon exposure to aqueous solution. As seen in Figure 1a, the Si2p core spectrum of silicon nitride could be deconvoluted into three peaks, which arose from [15]: N-Si-N (silicon nitride), N-Si-O (silicon oxynitride), and O-Si-O (silica) bond populations. As oxidation only affects the very surface of the material, the XPS probe, which is shallow at the single nanometer scale, was the most suitable probe to identify changes in surface chemistry. The N-Si-N peak component was preponderant on the pristine surface, but the surface exposed to water vapor environment showed a gradually increasing amount of O-Si-O bonds, as seen in the plot on the left side of Figure 1a. The increase in relative intensity of the O-Si-O peak occurred at the expenses of the N-Si-N one. This represents an unequivocal proof of oxidation with the formation of a silica layer. Note that the presence of silicon oxynitride bonds was not significantly altered up to 48 h. The zeta potential value at homeostatic pH of the Si_3_N_4_ surfaces was −50 mV and tended to slightly higher negative values with increasing autoclaving time concurrently with the increase in population of O-Si-O bonds.

The O1s core spectrum obtained by XPS measurement for the passive layer of the Ti6Al4V alloy substrate subjected to hydrothermal treatment, as seen in Figure 1b, was deconvoluted into four peaks according to Healy et al. [16,17] and Hierro–Oliva et al. [18]. The most pronounced peak, which can be seen at 531 eV, relates to oxygen atoms in the titanium oxide lattice; the peak at 532.9 eV belongs to the OH group. The Ti-O-Ti bridging oxygen bonds are proton-coordinated at the titanium oxide surface with oxygen from water molecules physically adsorbed on the titanium oxide surface. The peak displayed at 534.2 eV represents oxygen sites located at terminal Ti-OH bonds, for which the hydroxyl groups are chemically adsorbed on the titanium oxide surface. Finally, the peaks at 532.9 and 534.2 eV represent acidic and basic Ti-OH groups, respectively [19]. According to data in Figure 1b, a small population of both acidic and basic Ti-OH groups became present on the surface layer of the titanium alloy after hydrothermal treatment. The cumulative areal fraction of acidic and basic Ti-OH peaks remained small with increasing time of hydrothermal treatment. However, the presence of these peaks represented a strong indication that a small amount of anatase-type titanium oxide, which possesses both acidic and basic Ti-OH groups, formed on the surface of the titanium alloy substrate upon hydrothermal treatment. The most striking feature in the XPS spectrum of Ti6Al4V alloy subjected to water vapor environment was the rising peak centered at 528.1 eV, which can be assigned to adsorbed atomic oxygen [20]. Such an adsorbed oxygen species is known to be electron rich oxygen, which has been proposed to activate C–H bond breaking [21,22]. The presence of such highly reactive species could participate in the partial oxidation of surrounding molecules. Specifically, it is possible that unwanted reactions take place at the cell–substrate interface, which is a typically reported issue for adsorbed atomic oxygen [23]. The zeta potential value at homeostatic pH of the Ti6Al4V alloy surfaces was −20 mV.

The behavior of alumina surface during the accelerated test in water vapor environment is shown in Figure 1c. The Al2p core spectrum of alumina was deconvoluted into three peak components: hydroxylated (O-Al-O-H) bonds, non-hydroxylated (O-Al-O) bonds, and O-Al-V_O_ bonds representing the bond population at the material surface. A comparison between pristine and 24 h water-vapor-exposed samples revealed quick stoichiometric variations at the surface of the oxide substrate. A fraction of O-Al-V_O_ bonds gradually replaced both O-Al-O and O-Al-O-H bonds. Water molecules experience different strengths when hydrogen bonds to oxide and non-oxide ceramic surfaces (i.e., aluminols in Al_2_O_3_-based and silanols in Si_3_N_4_ ceramics) [24]. In the silanols developed on the Si_3_N_4_ surfaces, H-bond acceptors were strong while H-bond donors were weak. Conversely, in the aluminols developed on the Al_2_O_3_ surfaces, H-bond donors were relatively strong and acceptors relatively weak. The nature of H-bonds is key in the dual role of a surface as solvent of oxidant. The fundamental difference between the acid character of surface silanols and the basic one of aluminols has important repercussions on the metabolism of mesenchymal cells exposed to different bioceramic substrates. The zeta potential value at homeostatic pH of the Al_2_O_3_ surfaces was +20 mV.

### 2.2. Mesenchymal Cell Proliferation and Differentiation

**Figure 2:**
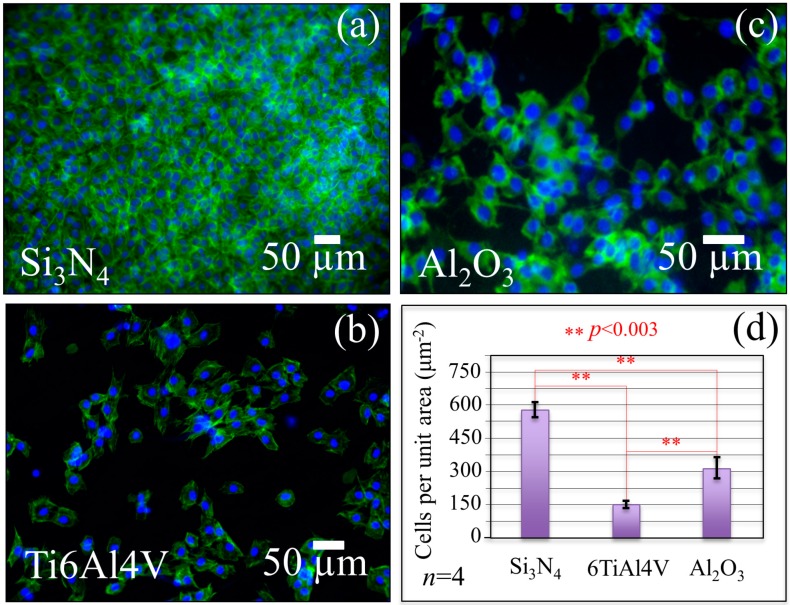
Fluorescence microscopy images of stained KUSA-A1 cells (nuclei and F-actin in blue and green, respectively) that proliferated for 24 h on (**a**) Si_3_N_4_, (**b**) Ti6Al4V alloy, and (**c**) Al_2_O_3_ substrates; in (**d**), quantitative plot and statistical validation of cell counting on different substrates. Statistics were made according to the one-way Analysis of Variance (ANOVA) to assess the plot in (**d**).

**Figure 3:**
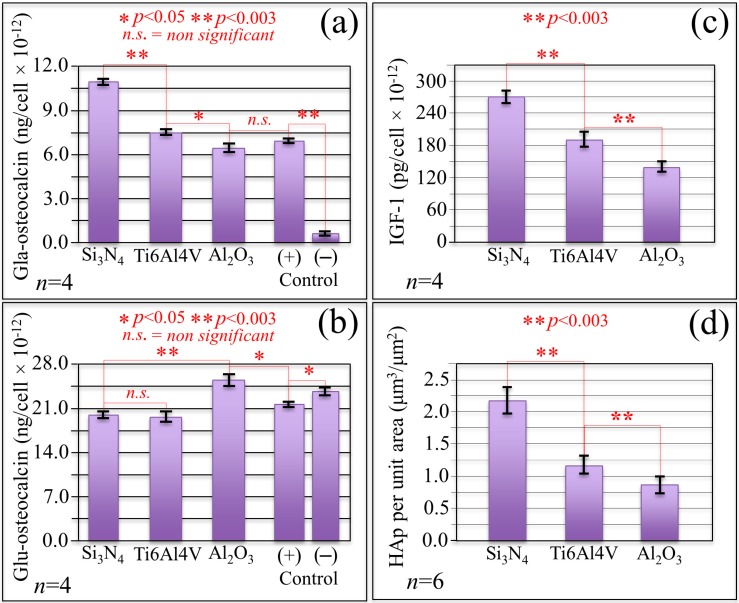
In (**a**) and (**b**), comparative plots of Gla- and Glu-osteocalcin, respectively, for cells exposed for nine days to different substrates; in (**c**), a similar evaluation is shown for IGF-1; (**d**) gives a plot of the volume per unit area of bony apatite grown by cells after exposure for 14 days to different substrates. Statistics were made according to one-way Analysis of Variance (ANOVA).

Figure 2 and Figure 3 summarize data collected by fluorescence microscopy and other biological assays, and laser microscopy data collected on KUSA-A1 mesenchymal cells exposed to different substrates. These figures also provide statistically data validations. The fluorescence micrographs in Figure 2 (nuclei and F-actin in blue and green, respectively) confirmed that the population of KUSA-A1 cells was clearly more numerous on the Si_3_N_4_ substrates after exposure times as short as 24 h, as seen in Figure 1a, while the number of cells was far lower on Ti6Al4V alloy substrates, as seen in Figure 1b, and relatively low on Al_2_O_3_ substrates, as seen in Figure 1c. The plot in Figure 2d quantitatively compares cell-counting data for 24 h exposure to different substrates. 

Assessments of differentiation and osteogenic metabolism in mesenchymal KUSA-A1 cells exposed for nine days to different substrates are given in Figure 3a–c. In Figure 3a,b, comparative plots of γ-carboxyglutamic (Gla)- and undercarboxylated (Glu)-osteocalcin, respectively, are given for cell-line exposure to different substrates. Statistically significant differences in concentration can be noticed in the Gla-osteocalcin plot, with the Si_3_N_4_ substrate reaching values more than one order of magnitude larger than and almost twice as those recorded for negative and positive control samples, i.e., cultured on silica glass without and with osteogenic medium, respectively, but otherwise under the same experimental conditions. The concentration of Gla-osteocalcin for mesenchymal cells exposed to the Si_3_N_4_ substrate was about 56% and 89% higher than those measured for exposures of cells to Ti6Al4V alloy and Al_2_O_3_ substrates, respectively. The Gla residue of osteocalcin is a specific and highly sensitive parameter for quantitative assessments of decarboxylated osteocalcin from the bony tissue produced by KUSA-A1 cells that differentiated into mature osteoblasts. Conversely, concentrations of the Glu residue, which is synthesized by osteoclasts, were the lowest in both Si_3_N_4_ and Ti6Al4V alloy substrates; no statistically significant difference between the two values was found but both were lower by ~40% and 30% than both positive and negative control, respectively. Note that the Al_2_O_3_ substrate experienced the lowest and the highest concentration of Gla- and Glu-osteocalcin supernatants, respectively, thus not only revealing the lowest propensity to osteoblastogenesis but also a conspicuous propensity for the mesenchymal cells to differentiate into mature osteoclasts.

Insulin-like growth factor 1 (IGF-1) signaling, as seen in Figure 3c, which can also be a probe for cell proliferation and differentiation efficiency, gave results comparable to those of the Gla-osteocalcin supernatant assay. The IGF-1 protein, which stimulates both differentiation and apatite growth, quantitatively probed a significantly higher metabolic propensity for the KUSA-A1 cells to differentiate into osteoblasts and subsequently generate more copious amounts of native apatite on Si_3_N_4_ when compared with Ti6Al4V alloy substrates. Conversely, the results of the IGF-1 assay in the plot of Figure 3c confirmed that mesenchymal cells cultivated on Al_2_O_3_ substrates possessed the lowest propensity to differentiate into mature osteoblasts. The IGF-1 supernatant concentration for cells exposed to the Al_2_O_3_ substrates was 93% and 32% lower than that detected for cell exposed to Si_3_N_4_ and Ti6Al4V substrates, respectively. The KUSA-A1 cells exposed to the Si_3_N_4_ substrate produced a IGF-1 concentration more than 300% higher than that of the positive control cell sample cultured on silica glass under the same conditions, not shown in the plot of Figure 3c. Figure 3d gives a quantitative estimate of the amount of bony apatite as obtained from laser microscopy assessments at the end of cell-culture experiments (14 days) on different substrates. As a conceivable consequence of the results collected by signaling assays at earlier stages of cell culture, the volumetric amount of bony apatite per unit area measured on Si_3_N_4_ substrates greatly exceeded those recorded for cultures on Al_2_O_3_ and Ti6Al4V substrates (170% and 87% higher, respectively).

**Figure 4:**
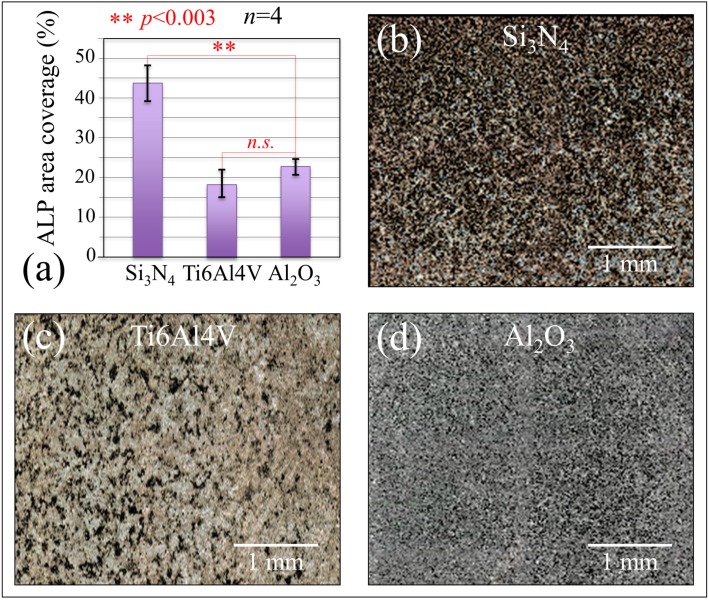
In (**a**), quantitative assessment of ALP concentration as obtained from stained micrographs on Si_3_N_4_ (**b**), Ti6Al4V (**c**), and Al_2_O_3_ (**d**).

Figure 4a shows the results of alkaline phosphatase (ALP) staining while micrographs showing the black ALP stain on the Si_3_N_4_, Ti6Al4V, and Al_2_O_3_ substrates at nine-day exposure time are shown in Figure 4b–d, respectively. The ALP content of cells cultured on the Si_3_N_4_ substrate was about twice that of cells cultured on the Al_2_O_3_ substrate under the same conditions; cells on the Ti6Al4V substrate showed the lowest ALP concentration among the investigated samples (~20% lower than cell on Al_2_O_3_). However, there was no statistical difference between the ALP contents measured in cells cultured on these two latter substrates. ALP is an enzyme that represents cell differentiation and the present results prove that the KUSA-A1 cells differentiated faster on the Si_3_N_4_ substrate, a result in agreement with the IGF-1 results in Figure 3c.

### 2.3. In Situ Raman Analyses

**Figure 5:**
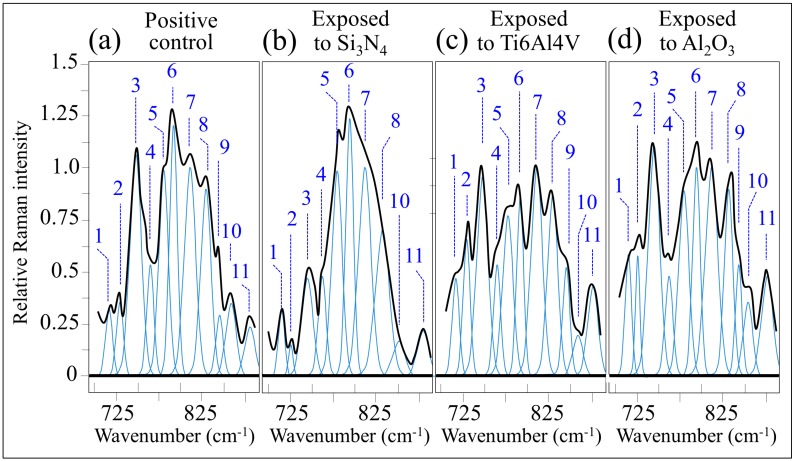
Low-frequency Raman spectra for (**a**) positive control cell sample, (**b**) cells exposed for 48 h to Si_3_N_4_ substrate, (**c**) cells exposed for 48 h to Ti6Al4V alloy substrate, and (**d**) cells exposed for 48 h to Al_2_O_3_ substrate. All the spectra were normalized to Band 7 (at 813 cm^−1^, related to symmetric stretching of the phosphodiester bonds of RNA [25,26,27]).

Figure 5 shows the low-frequency Raman spectra collected in situ for: (a) the positive control cell sample (cultured on silica glass substrate for 48 h with addition of osteogenic medium), (b) after 48 h culture on Si_3_N_4_ substrate, (c) after 48 h culture on Ti6Al4V alloy substrate, and (d) after 48 h culture on Al_2_O_3_ substrate. 

Eleven Raman bands were located in the monitored spectral window, the precise frequencies of which as well as their physical origins have been reported in a previous paper [10]. In the case of cells cultured on Si_3_N_4_ substrates, no significant variations in relative intensity when compared with the positive control sample could be observed after 48 h for RNA/DNA-related Bands 5 (at 783 cm^−1^), 6 (at 794 cm^−1^), and 7 (at 813 cm^−1^). These bands represented vibrations of phosphodiester symmetric stretching in DNA, ring breathing in cytosine, and phosphodiester symmetric stretching in RNA, respectively [25,26,27]. An exception among nucleic acid related bands was Band 8 (out-of-plane ring breathing in tyrosine [28]), which showed a clear decrease of approximately 20% in relative intensity in the spectrum collected on cells exposed to Si_3_N_4_ substrates in compared with control, as seen in Figure 5a,b. Conversely, the decrease in cytosine band was by far less pronounced in the case of cells cultured on both Ti6Al4V and Al_2_O_3_ substrates, as seen in Figure 5c,d, respectively. The decrease in relative intensity of Band 8, which reflects a reduction in tyrosine content, was consistent with the observation of a concurrent decrease in intensity of Band 10 (at 852 cm^−1^) related to tyrosine (benzene ring breathing [29]) but also contributed by O-P-O stretching in adenosine triphosphate. A striking additional feature in the spectrum of Si_3_N_4_-exposed cells when compared with the control was a dramatic decrease of the tryptophan Band 3 (at 748 cm^−1^) [26]. This feature could neither be detected in the Ti6Al4V nor in the Al_2_O_3_ substrates, as seen in Figure 5b,c, respectively. Other characteristics peculiar to the spectrum of mesenchymal cells exposed to Si_3_N_4_ substrates were: (i) the disappearance of a sharp shoulder Band 9 (at 843 cm^−1^) mainly related to phosphatidylinositol [30]; (ii) a clear decrease in relative intensity (and shift from 733 to 726 cm^−1^) of Band 2 (at 733 cm^−1^), which is a composite signal of phosphatidylserine (C-N stretching in the serine residue [30]) and adenosine triphosphate (adenine ring breathing [31,32]). The above variations of Bands 8, 10, 3, 9, and 2 are clear fingerprints of an altered cell metabolism that have only occurred upon short-term exposure to the Si_3_N_4_ substrate. The sacrificial reductions in the selected tryptophan purine, adenosine triphosphate, and protein tyrosine, as well as the clear alteration in phospholipids’ composition, i.e., disappearance of the phosphatidylinositol band and reduction in phosphatidylserine, are clear signs of osteoblastogenesis. Evidences obtained through comparative data on control samples and samples exposed to different substrates are also consistent with the thesis of an enhanced propensity to differentiation into osteoblasts as a consequence of cell–substrate interactions at the molecular scale. In particular, the decrease in relative intensity of the tyrosine ring breathing Bands 8 and 10 is a consequence of nitration in tyrosine proteins by NO radicals [33]. During osteoblastogenesis, the so-called kynurenine pathway is activated [34]. In this pathway, cells utilize tryptophan to synthesize proteins after cleaving its indole ring [35]. Tryptophan is a precursor in the synthesis of melatonin, which increases differentiation of human mesenchymal stem cells into the osteoblastic cell lineage [36]. Moreover, the reduced intensity of Band 2, to approximately a third of that of the control cell sample, and its shift toward lower frequencies can be interpreted as the spectroscopic fingerprints of deprotonation from -NH_3_^+^ to -NH_2_ in the serine group of the phosphatidylserine lipid structure of the cell membrane. Under the effect of alkaline phosphatase, calcium concentration increases in the cytoplasmic space, and Ca^2+^ ions binds to the COO^−^ terminus of the deprotonated serine residue forming Ca complexes [37]. Regarding the effect on other membrane lipids, the disappearance of the phosphatidylinositol Band 9 relates to the interaction between nitrogen radicals eluted from the Si_3_N_4_ substrate and the phosphate group of the inositol structure via transfer of H^+^ protons. Conversely, the structure of phosphatidylcholine (represented by Bands 1 and 11 at 718 and 876 cm^−1^, respectively [30]) remains unaffected because the choline ring in this phospholipid is neutral in nature. The chemical aspects of the interactions between mesenchymal cells and the Si_3_N_4_ substrate will be discussed in detail in the next section.

Despite some similarities with the spectrum of the control cell sample, distinct spectral features could be observed in the Raman spectra of mesenchymal cells exposed to Ti6Al4V and Al_2_O_3_ substrates, which could not be observed for cells cultured on Si_3_N_4_ substrates. In the spectrum of cells exposed to Ti6Al4V and Al_2_O_3_ substrates, the relative intensities of tryptophan-contributed Bands 3 and 4 remained comparable to that of the corresponding bands in the spectrum of the control sample. On the other hand, signals related to lipids (i.e., the Bands 1, 2, 9, and 11 contributed by C-N stretching in choline, C-N stretching in serine, cumulative signals from phosphatidylcholine and phosphatidylinositol, and C-N antisymmetric stretching in choline, respectively) experienced an increase in their relative intensities. Conversely, the two DNA-related Bands 5 and 6 (seen at 783 and 794 cm^−1^, respectively) were of lower relative intensities when compared with the control cell sample and to the cell sample exposed to Si_3_N_4_ substrate, whose 5 and 6 signals were similar to those in the control; however, such intensity reductions were slightly more pronounced for the case of Ti6Al4V than for Al_2_O_3_ substrates. In a recent paper, Brauchle et al. [38] monitored the Raman spectra of apoptotic osteosarcoma and chondrosarcoma cell lines *in situ*. For both cell lines, the DNA signals at approximately 780~800 cm^−1^ decreased during apoptosis. The authors suggested that such a decrease was related with to progress of internucleosomal DNA cleavage. Analogous to that study, one could also interpret the spectral changes observed in the Raman spectra of the present KUSA-A1 mesenchymal cells exposed to both Ti6Al4V alloy and Al_2_O_3_ substrates as fingerprints of intracellular biochemical changes correlated to toxic challenges [39]. The exogenous development of reactive oxygen species (ROS) at the biomolecular interface between cells and substrate could be one possible cause of irreversible damages to proteins and nucleic acids as well as the peroxidation of membrane lipids, which can lead to activation of cell death processes such as apoptosis [40]. The chemical origin of these processes detrimental to cell proliferation and osteoblastic activity will be discussed in detail in the next section.

## 3. Discussion

### 3.1. Off-Stoichiometric Reactions at the Cell–Substrate Interface

**Figure 6:**
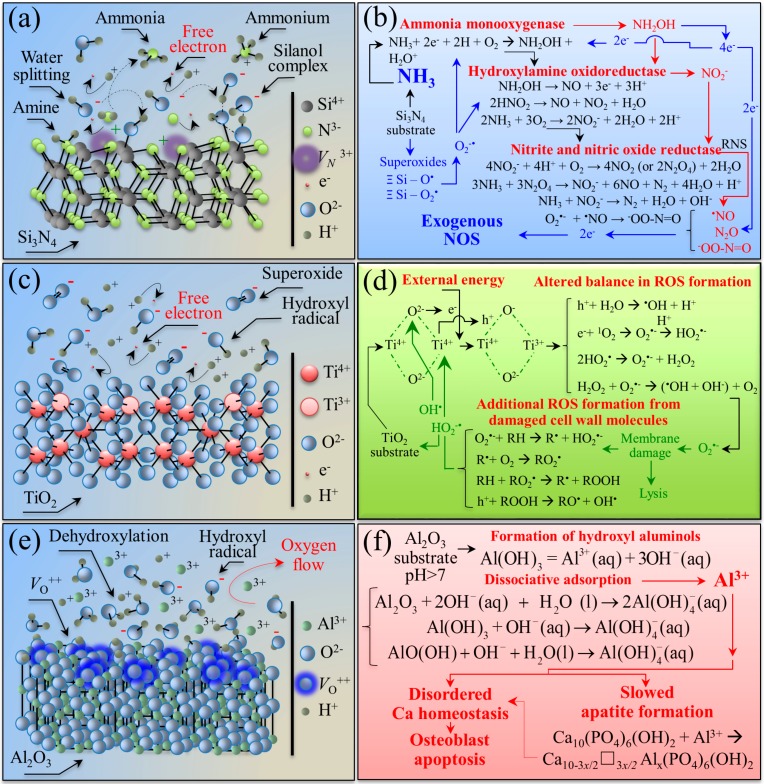
Schematic drafts of the free surface of different substrates in aqueous environment (Si_3_N_4_, Ti6Al4V alloy, and Al_2_O_3_ in (**a**,**c**,**e**), respectively) and the cascade of related off-stoichiometric reactions (Si_3_N_4_, Ti6Al4V alloy, and Al_2_O_3_ in (**b**,**d**,**f**), respectively).

Figure 6 shows schematic drafts of the free surface of the investigated substrates and the set of off-stoichiometric reactions involved with their embedment in an aqueous solution. 

The drafts and the reactions listed in the figure are based on the results of accelerated tests in water vapor provided in Section 3.1 and works published by these and other authors [41,42,43,44,45,46,47]. The behavior of Si_3_N_4_ bioceramic is described in Figure 5a,b. In aqueous solution, the surface lattice of this non-oxide ceramic is subjected to dissociation of its Si-N covalent bond. Such a homolytic bond cleavage liberates nitrogen ions to the environment, creates a nitrogen vacancy V_N_^3+^, and exposes silicon sites on the free surface. The former ions immediately join hydrogens in solution to form free ammonium ions NH_4_^+^ or ammonia NH_3_; simultaneously, the latter ions find a new equilibrium state upon linking to OH ions to form silicon dioxide SiO_2_ that further reacts with water and produces stable silanol structures Si(OH)_4_ on the bioceramic surface, as seen in Figure 6a. A straightforward outcome for the elution of nitrogen and the consequent formation of NH_4_^+^/NH_3_ at the solid–liquid interface is a robust enhancement of local pH [48,49]. XPS data in accelerated water vapor environment confirmed this tendency, as seen in Figure 1a, showing increasing amounts of N-Si-O and O-Si-O bond populations at the expenses of the N-Si-N one. An important aspect of the hydrolysis process at the Si_3_N_4_ surface is that dissociation of both surface amine and silanol sites eventually generates free electrons and oxygen/nitrogen free radicals [41,42,43]. The off-stoichiometric chemical reactions of the newly formed species with the available free electrons ultimately lead to the formation of NO radicals, which have been experimentally observed and measured *in situ* in a previous work [44]. The cascade of off-stoichiometric reactions shown in Figure 6b can be explained with the dissociation of surface silanols as the initial step in the formation of superoxide radicals (≡ Si – O^•^) and (≡ Si – O^•^_2_). Unpaired electrons can then react with adsorbed O_2_ to form O_2_^−^^•^ radical anions or other protonated radicals with highly oxidative characteristics. These species could, in turn, react with NH_3_, leading to its oxidation into hydroxylamine (NH_2_OH), according to a process referred to as ammonia monooxygenase. The availability of two free electrons from ammonia enables additional reductant contributions, according to which the second O atom forms H_2_O and leads to further oxidation of NH_2_OH into nitrite (NO_2_^−^). This process, which is referred to as hydroxylamine oxidoreductase, releases four electrons, two of which can promote further ammonia oxidation. Note that, in eukaryotic cells, the mitochondrion is also a major source of oxygen radicals with the transfer of four electrons to an O_2_ molecule made possible through cytochrome c oxidase enzymatic catalysis. However, this process is accompanied by the eventual formation of byproducts of partially reduced species as a consequence of leakage from transport chains of the electrons associated with the transfer [45]. The availability of the leaked free electrons can also serve to neutralize small infiltrations of exogenous NH_3_ through the cell plasma membrane by oxidizing it into hydroxylamine NH_2_OH. Additional gaseous reactive nitrogen species (RNS) products of NH_3_ oxidation are NO and ^−^OONO, which are also highly reactive and oxidative species [46]. The successive off-stoichiometric events of monooxygenase, oxidoreductase, and the formation of gaseous oxidizing byproducts originate from the concurrent presence of ammonia and silanols, and are thus peculiar to the Si_3_N_4_ surface in aqueous environment. Among the exogenously produced RNS at the biomolecular interface between cells and the Si_3_N_4_ substrate, NO covers a fundamental role in cell signaling. Specifically regarding the topic of the present work, as a reactive byproduct of ammonia and ammonium, exogenous nitric oxide NO can participate in enzymatic reactions and displace osteoblastogenesis and glutamine synthetase equilibriums [47]. Note that the elution of nitrogen and the successive formation of NH_4_^+^/NH_3_ locally enhance pH at the cell–substrate interface, as experimentally demonstrated in previous papers [48,49]. Such a pH buffering process boosts alkaline phosphatase (ALP) with calcium concentration locally increasing and Ca^2+^ binding to the COO^−^ terminus of the deprotonated serine residue to form Ca complexes. This interpretation fits both the present ALP results (Figure 4) and the lipid structural alterations recorded in the Raman data of Figure 5a.

Figure 6c,d give a schematic draft of the free surface of the Ti6Al4V alloy substrate and the set of off-stoichiometric reactions involved with its embedment in aqueous solution, respectively. A previous study by Jennison et al. [50] has shown that a TiO_2_ passivation layer naturally forms on Ti6Al4V alloy. One reason why the Ti6Al4V alloy is widely used in biomedical implants is that its TiO_2_ surface layer possesses antibacterial efficacy, a property exploited in advanced biomedical technologies [51]. The chemical circumstance behind this property is the free-radical effect of the passivation TiO_2_ layer [52,53,54]. However, despite the wide attention given to the antibacterial properties of titanium alloys, studies on the effect of their surface chemistry on cells have seldom been published. 

The TiO_2_ surface of Ti6Al4V alloy can change into TiOH metastable intermediates, as seen in Figure 6c, a process that is greatly enhanced by ultraviolet light irradiation but can partly take place also in dark environments [55]. The origin of the charge at the TiO_2_ surface resides in the formation of oxygen vacancies consequential to a reductive change in valence from Ti^4+^ to Ti^3+^ [56]. Figure 6d summarizes the oxygen radical chemistry pathways at the surface of the TiO_2_ passivation layer. Electrons e^−^ and holes h^+^ are generated which lead to dissociative chemisorption of water from the environment. In this study, the occurrence of the chemisorption process has directly been observed through XPS analyses, as seen in Figure 1b. The consequence of water chemisorption is the release of reactive oxygen species (ROS), such as hydroxyl radicals (^•^OH), superoxide (O_2_^−^^•^), singlet oxygen (^1^O_2_), and hydrogen peroxide (H_2_O_2_) from the TiO_2_ substrate [57]. The generated holes and electrons, precursors to the formation of ROS, recombine with free electrons to form new O_2_^−^^•^ and H_2_O_2_. At the interface with prokaryotic cells, such highly oxidative radicals interact with the membrane species to produce degraded species and additional ROS. Unlike the relatively less active O_2_^−^^•^ and H_2_O_2_, which can be detoxified through both enzymatic and non-enzymatic endogenous antioxidants, no enzyme can detoxify ^•^OH and O_2_^−^^•^, and they induce extremely acute effects on bacteria [45,58]. However, the same circumstances that render ROS acutely lethal to bacteria might also lead to self-propagating chain reactions that ultimately lead to cell lysis. In this study, *in situ* collected Raman data on mesenchymal cells exposed to Ti6Al4V alloy substrates suggest molecular interactions at the cell–substrate interface that are not of a friendly nature. Spectroscopic features point at DNA damage and metabolic reactions in membrane lipids as fingerprints of incipient apoptosis induced by ROS.

Figure 6e,f show a schematic draft of the free surface of the Al_2_O_3_ substrate in aqueous environment and a set of related off-stoichiometric reactions, respectively. The surface of Al_2_O_3_ contains both acid and basic sites depending on the termination of the bulk surface, namely the exposure of metal cations for Lewis acid sites or oxygen atoms for Lewis and Brønsted basic sites. Minimization of the surface free energy takes place upon producing terminations with hydroxyl groups. Pure alumina contains only one type of cation and the hydroxyl groups at its surface are expected to be weakly to moderately acidic (due to the high ionicity of the Al–O bond) and, thus, to form weak hydrogen bonds with sorbed molecules. Hydroxyl groups might eventually be removed from the alumina surface upon thermal-frictional activation, and coordinatively unsaturated (oxygen) anions and exposed Al^3+^ cations (and anion vacancies, V_O_^2+^) are formed on the surface. The formation of oxygen vacancies at the expenses of O-AlO bonds has been observed in this study by XPS analyses applied to substrate subjected to accelerated thermal activation, as seen in Figure 1c. In this context, the removal of OH groups happens concurrently with the development of OH site basicity, because the net positive charge at the anion vacancy becomes lower as the net negative charge of the removed OH group rises. Proton acidity and OH group removal are key factors in governing dehydroxylation, and they play a fundamental role on the “degree of bioinertness” of the oxide bioceramic surface, as seen in Figure 6e. Note that the coordinatively unsaturated Al ions represent strong electron acceptors (Lewis acid) sites for dissociative adsorption of water [59]. This process involves charge transfer from the Al_2_O_3_ cluster to hydroxyl, oxygen, and proton radicals, and is more energetically favorable than molecular adsorption, in which H_2_O donates its charge to the Al_2_O_3_ cluster [60,61]. Figure 6f shows the cascade of events taking place at physiological pH on an Al_2_O_3_ substrate. The process starts with the formation of hydroxyl aluminols, eventually followed by dissociative adsorption of the aluminols to liberate Al^3+^ and OH^−^ ions in solution. The removal of hydroxyl groups involves the formation of oxygen vacancies, with the OH groups being easily removed as a consequence of OH site basicity (i.e., the net remaining positive charge at anion vacancy site is lower than the net negative charge on the removed OH group). There are two consequences of dissociative adsorption at the Al_2_O_3_ surface. On the one hand, Al^3+^ inhibits mineralization by slowing the rate of hydroxyapatite crystal formation, as seen in the chemical equations for the Al-modified apatite structure in Figure 6f, and altering the influx and efflux of calcium from bone cells [62]. On the other hand, the presence of Al^3+^ could lead to oxidative stress through the formation of ROS with a significant perturbation of the normal anti-oxidative system [63]. Chambers and LoGrasso [64] reported *in vivo* and *in vitro* data showing that Al-induced oxidative stress activates cell apoptosis signaling pathways. The induction of oxidative stress by ROS is one of the toxic effects involved with the exposure of cells to aluminum, and its consequences include irreversible modifications of cellular proteins, lipids, and DNA [65]. A recent *in vitro* experimental study by Li et al. [66], which specifically targeted the oxidative injuries induced by aluminum to osteoblasts, showed that the osteoblast apoptosis occurred within 24 h exposure and its rate increased with increasing the aluminum dose (in the order of 10^−2^ mg/mL). Membrane lipid rigidification occurs through chemical interaction between Al^3+^ ions and the phosphate groups of phospholipids, which greatly limits cell mobility. The results were thus straightforward in proving that Al exposure causes significantly oxidative stress to induce apoptosis and injuries in osteoblasts. The results of the present experiments are in line with the above-cited studies. Although the kinetics of dissociative adsorption at the Al_2_O_3_ surface should be much slower in terms of aluminum elution than the direct doping experiments proposed in Refs. [62,63,64,65,66], yet we observed significant inhibition in osteoblastogenesis and a very low propensity to deposit bony apatite on Al_2_O_3_ when compared with other substrates. Remarkably, also *in situ* Raman experiments conducted after short-term exposure of mesenchymal cells to Al_2_O_3_ substrates provided molecular scale fingerprints, which could be interpreted as an apoptotic tendency of the mesenchymal cells.

### 3.2. RNS Formation and the Cell-Friendly Kinetics of Silicon Nitride

In mesenchymal cells, the isoenzymes referred to as endothelial (eNOS) and inducible (iNOS) are responsible for the production of NO, a short-lived and extremely volatile molecule that regulates both cell survival and death. Capable of freely passing through the cell membrane, NO is an intercellular messenger whose message is internalized through guanylate cyclase, a lyase enzyme that regulates the intracellular calcium level [67]. At low concentrations, NO is used by cells for signaling enhanced proliferation; however, in high concentrations, it causes apoptosis. In the clinical practice, NO can be generated, irrespective of production in the cells, by exploiting synthetic compounds such as sodium nitroprusside. This exogenous donor produces NO upon reacting with sulphydryl groups of the erythrocyte membrane, albumin, and other proteins via non-enzymatic and enzymatic processes [68]. Extensively used as a venous vasodilator, sodium nitroprusside serves to immediately decrease the blood pressure in critical clinical situations [69]. The efficacy of exogenous NO on the *in vitro* osteogenic differentiation of mesenchymal stem cells has been the object of a recent study by Abnosi and Pari [70]. These researchers discovered that low concentrations of exogenous sodium nitroprusside (in the order of <0.1 mM generating ~1.2 nM of NO) promoted the ability to proliferate and the capacity to differentiate into osteoblasts of rat bone marrow mesenchymal stem cells. This effect was attributed to an up-regulation by NO of alkaline phosphatase, the critical enzyme that releases the phosphate and causes calcium ions to enter the cells and form apatite. Conversely, concentrations in the order of 1 mM significantly reduced cell viability, an effect also contributed to by the concurrent release of cyanide and iron as toxic byproducts of NO synthesis from exogenous sodium nitroprusside compound. Cyanide is a toxic compound that inhibits cytochrome c oxidase in the cell respiratory chain [71]; inorganic iron has also been reported to inhibit the process of mineralization [72]. Sodium nitroprusside also generates ROS, which induce lipid peroxidation and cytotoxicity [73].

**Figure 7:**
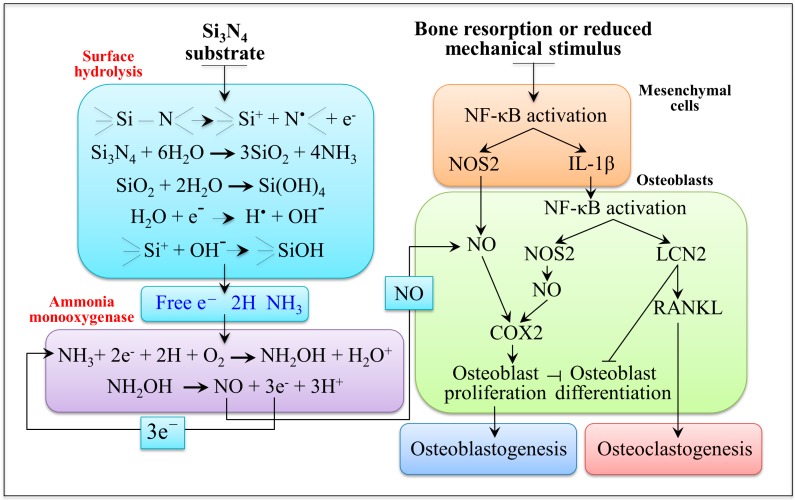
Schematic representation of the pathways involved with osteoblast-osteoclast crosstalk (IL-1β/LCN2/RANKL pathway leading to osteoclastogenesis and NOS2/NO/COX2 pathway leading to osteoblastogenesis); an exogenous NO uplift represents the final effect of RNS formed at the cell/Si_3_N_4_ interface as a consequence of hydrolysis and the subsequent cascade of chemical reactions taking place on the Si_3_N_4_ substrate in aqueous environment.

One of the key findings of the present study was the promotion of both proliferation and osteoblastogenesis of mesenchymal KUSA-A1 cells, which appears to be greatly enhanced when the cells are exposed to Si_3_N_4_ substrates. Biological assays probing for cell proliferation, as seen in Figure 2, differentiation, as seen in Figure 3a–c, and bony apatite production, as seen in Figure 3d, unequivocally showed a consistent picture of up-regulated osteogenesis as a consequence of the peculiar surface chemistry of Si_3_N_4_ bioceramics. During differentiation, mesenchymal cells utilize both oxidative phosphorylation and glycolysis pathways [74]. From a biochemical point of view, the initially high adenosine triphosphate level tends to linearly decrease upon cell differentiation in favor of increased diphosphate and monophosphate levels because released phosphate groups are needed for reaction with Ca^2+^ to synthesize hydroxyapatite crystals [75]. A decrease in bands contributed by adenosine triphosphate, which reflects a decreased intracellular concentration of this compound, was observed by *in situ* Raman spectroscopy, as seen in the decrease in Bands 2 and 10 in Figure 5b in comparison with the control sample in Figure 5a. The exogenous production of NO at the cell/Si_3_N_4_ interface has been explained in Figure 6a,b, according to a cascade of reactions originated from surface hydrolysis and including ammonia monooxygenase, hydroxylamine oxidoreductase, and nitrite/nitric oxide reductase. According to the obtained results, the NO amount released from the Si_3_N_4_ substrate appears to be low and slow enough to fall into the concentration range within which it operates as a signal for enhancing proliferation. A further important characteristic of the exogenous NO production from a Si_3_N_4_ surface is the absence of any toxic byproduct, different from the case of cyanide and iron ions released by the sodium nitroprusside donor. The only ionic byproduct of Si_3_N_4_ surface hydrolysis (beside nitrogen) is silicon, which is also an element that gives a positive contribution to osteogenesis [76,77]. (SiO_4_)^4−^ tetrahedra and Si nanodots were found in the structure of bony tissue grown both *in vitro* and *in vivo* by human osteoblasts in proximity of Si_3_N_4_ surfaces [44,78]. With regard to the differentiation of mesenchymal cells, osteoblast and osteoclast functions are regulated by the so-called NF-κB, a set of multifunctional transcriptional factors controlling the expression of the genes involved with skeletal development (among numerous other cellular activities) [79]. Figure 7 gives a schematic draft of NF-κB activation in response to a state of bone resorption or as a consequence of a reduced mechanical stimulus. 

There are two distinct NF-kB pathways that lead to either osteoclastogenesis or osteoblastogenesis. The mechanisms according to which NF-κB can induce osteoclastogenesis downstream of receptor activator of nuclear factor-kappa B ligand/receptor activator of nuclear factor-kappa B (RANKL/RANK) are not yet completely understood. According to a recent study by Veeriah et al. [80], the osteoclastogenesis pathway could be described as follows: the pro-inflammatory cytokine IL-1β becomes active upon cleavage by caspase-1 and acts a pro-osteoclastogenic factor [80,81]. Ineffective in osteoblast proliferation, but active in reducing their differentiation, the pleiotropic and acute-phase protein Lipocalin 2 (LCN2) is upregulated by inflammation and its induction produces significant osteoblast impairment. LCN2 in turn stimulates RANKL production by osteoblasts, which favor osteoclastogenesis and bone resorption. Parallel to the IL-1β/LCN2/RANKL pathway, NF-kB activation also induces NOS2, which increases osteoblast proliferation through the formation of NO molecules that downstream target Cyclooxygenase-2 COX2 (NOS2/NO/COX2 pathway). Maintaining the balance between these two pathways is key for the metabolism of healthy bone. We have shown here that the Si_3_N_4_ surface could become an exogenous NO donor in aqueous solution as a consequence of the homolytic cleavage of its covalent Si-N bonds. NO molecules are generated from ammonia and its subsequent monooxygenase by means of free electrons made available by both silanol dissociation and by partially reduced oxygen radical byproducts that form as a consequence of leakage from transport chains of cytochrome c oxidase enzymatic catalysis in the mitochondrion. Such an amount of exogenously produced NO is expected to boost the NOS2/NO/COX2 pathway and to promote osteoblastogenesis, as seen in Figure 7. In a recent paper, Shen et al. [13,82] found that pH values >8 at the cell–substrate interface greatly enhance both cell proliferation and alkaline phosphatase activity of osteoblasts. This study is important because it shifts the focus in osteogenesis to the chemistry of the solid–liquid interface as a critical factor in bone regeneration. We have previously reported [48,49] that the interfacial pH at the cell/Si_3_N_4_ interface is locally enhanced and stabilized around a value ~8.25. This may be of importance in explaining the enhanced activity of mesenchymal cells on the Si_3_N_4_ surface; alkalosis is known to promote bone formation [83] while systemic acidosis promotes bone resorption [84,85,86].

In conclusion, the good agreement described in Section 2.2 among Gla/Glu-osteocalcin assays, IGF-1 assay, ALP assay, and quantitative laser microscopy volumetric assessments of bony apatite deposited by cells after 14 days of culture provided proof that cell differentiation into mature osteoblasts, production of osteocalcin binding onto apatite, and successive growth of bony tissue were greatly enhanced when mesenchymal progenitor cells became exposed to Si_3_N_4_ substrates. Moreover, the decreases in tryptophan and phospholipids Raman signals observed in the Raman spectrum of mesenchymal cells exposed to Si_3_N_4_ substrates provide spectroscopic fingerprints of osteoblastogenesis. This interpretation of the *in situ* Raman data is consistent with the outputs of biological assays, as seen in Figure 3, for cells exposed to Si_3_N_4_ substrate. On the basis of our present findings, the Si_3_N_4_ surface chemistry with its interfacial pH buffering effect and friendly RNS kinetics might be exploited in counteracting bone resorption pathologies, in promoting bone repair, and in many related contexts in bone-tissue engineering.

### 3.3. ROS Formation and the Issue of Bone Resorption

The biomedical grade Ti6Al4V alloy substrate tested in this study showed the least cell proliferation rate among the tested substrates, as seen in Figure 2. However, the concentrations of Gla-osteocalcin and IGF-1 supernatants, which characterize the propensity to differentiate into osteoblasts, were slightly higher than that of cells exposed to Al_2_O_3_ substrates, as seen in Figure 3a,c. An explanation for the observed trends could be that, although the metal ions eluted from the surface of the alloy disrupt cell proliferation, the isolated groups of mesenchymal cells that succeed in surviving and differentiating into osteoblasts deposit a protein layer at the solid interface, which eliminates a direct metal/cell interface. This explanation is supported by the “island-like” morphology and the osteopontin-rich composition of bony apatite observed for *in vitro* osteoblast cultures on Ti6Al4V alloy substrates [10,87]. Biocompatibility is generally related with the corrosion property of the metallic alloy and its surface treatments because metal ions can be released into the adjacent environment during the corrosion process affecting the periprosthetic tissues [88,89,90,91].

Thompson and Puleo [92] were the first to spot the metal ions released from Ti6Al4V implant components as potentially disturbing agents of the normal functions of bone cells. Such ions stimulate bone resorption by osteoclasts or inhibit bone synthesis by osteoblasts. To prove their hypothesis, these researchers exposed osteogenic cells to metal ions and studied the short-term (48 h) effects of Ti^4+^ and A1^3+^ ions on the osteoblastic phenotype [93]. These pioneering studies determined toxicity of such metal ions, which caused gross cell death. The seriously deleterious effect of Ti^4+^ and A1^3+^ ions on mesenchymal cell cultures at physiologically relevant concentrations was attributed to the interference by these ions with cell differentiation into mature osteoblasts. According to *in vitro* data, these researchers predicted that the interference of metal ions associated with Ti6Al4V implants with normal osteoblastic differentiation could decrease normal *in vivo* bone deposition and contribute to implant failure by impairing bone repair. This describes the current situation, which is partly observed for Ti6Al4V components of dental implants according to the most recent official statistics [94]. From the point of view of the osteogenic behavior of Ti6Al4V alloys, the present *in vitro* study does not add new data to the published literature. However, we report here a slow rate for mesenchymal cell proliferation and differentiation into osteoblasts, as seen in Figure 2 and Figure 3a,c, accompanied by a clear propensity to differentiate into osteoclasts, as seen in the Glu-osteocalcin data in Figure 3b. Moreover, the volumetric amount of bony apatite produced in 14 days by the present cell culture on Al_2_O_3_ substrates was the least among the tested substrates, as seen in Figure 3d. Al_2_O_3_ is not cytotoxic to human cells and is generally considered as a fully bioinert biomaterial [1,2,3], which may be a fair statement when this oxide bioceramic is compared with metallic materials. However, the present study clarifies that the Al_2_O_3_ surface does not represent a friendly environment for mesenchymal cells. In addition, we explain the poor outputs of Al_2_O_3_ in osteogenic performance as a consequence of its non-bioinert surface chemistry, as seen in Figure 6e,f. A dehydroxylation reaction leading to the formation ROS, including hydroxyl, and liberated Al^3+^ ions in aqueous solution might induce lipid rigidification/peroxidation, delay osteoblastogenesis, and inhibit the effect of mineralization. We have also put forward spectroscopic proofs for a propensity to apoptosis for mesenchymal cells exposed to Al_2_O_3_ substrates, which was comparable with that of cells exposed to those of Ti6Al4V, as seen in Figure 5c,d. The decrease in DNA Raman bands and the increase in bands related to lipids, described in Section 2.3, are fingerprints of incipient apoptosis induced by ROS and metal ions (as generated in different concentrations) on Ti6Al4V alloy and Al_2_O_3_ substrates. The kinetics of dehydroxylation and ionic elution from the Al_2_O_3_ surface might be quite slow under static conditions at physiological pH. However, the ionic elution kinetics could significantly be accelerated under frictional conditions or in an acidic environment. In view of its poor osteogenic performance and because of the above reasons, components made of Al_2_O_3_ bioceramics might not be suitable for implants subjected to friction or highly localized stresses in the human body; they may also be prone to infections. Accordingly, we believe that Si_3_N_4_ non-oxide bioceramics are more suitable than oxide bioceramics, despite a number of publications have suggested the use of Al_2_O_3_ and Al_2_O_3_-based biomaterials as components for dental and spinal fusion implants [95,96,97,98,99]. Components for these biomedical applications, which require a quick, massive, and long-term protracted osteogenesis, should be designed not only in consideration of the structural reliability of the bioceramic [100] but also of their short- and long-term osteogenic attitudes.

### 3.4. Possible New Applications and the Future of Si_3_N_4_ Bioceramics

The focus of the present study was to present a detailed explanation of the physical chemistry circumstances behind the friendly interaction and kinetics of Si_3_N_4_ toward osteoblasts. In particular, we targeted the positive contribution of this non-oxide material as an exogenous NO donor in osteoblastogenesis. On the one hand, this finding clearly strengthens the suitability of Si_3_N_4_ bioceramic in spine arthrodesis, which is currently its primary application as a biomedical component. On the other hand, it also paves the way to establish new biomedical applications for this material, in which the balance between osteoblast and osteoclast activity, which governs bone turnover, needs to be restored. For example, Si_3_N_4_ could be used as bulk components in various arthroplastic applications (also in view of its excellent mechanical properties [100]) or as a coating to metallic stems and acetabular components of hip joints to limit any long-term contact between metal surfaces and bone tissue. The use of Si_3_N_4_ components could be particularly useful in primary surgeries on patients affected by osteoporosis or for those patients for whom the primary surgery has produced osteolytic bones. The paradigmatic shift in surgeries based on the Si_3_N_4_ approach would thus potentially consist of using a “therapeutic” rather than a passively inert biomaterial for artificial implants. Exploiting the long-term supportive (chemical) driving force and the friendly kinetics of Si_3_N_4_ bioceramics will not only elongate the in-service lifetime of implanted components but also restore the fundamental function of osteoblastogenesis in patients affected by osteoporosis or by other degenerative changes associated with aging. In this context, the availability of a “therapeutic” (rather than inert) biomaterial with long-term kinetics efficacy such as Si_3_N_4_ could represent an important option in societies with an increasing elderly population.

## 4. Materials and Methods 

### 4.1. X-ray Photoelectron Spectroscopy

Dense Ø12.7 mm × 1 mm and finely polished discs of silicon nitride (Si_3_N_4_, Mc^2®^, SINTX Corporation, Salt Lake City, UT, USA), titanium alloy (Ti6Al4V, ASTM F136), and medical grade alumina (Al_2_O_3_, BIOLOX®Forte, CeramTec GmbH, Plochingen, Germany) were prepared. XPS experiments were then carried out on the surfaces of the different substrates to characterize their surface chemistry before and after exposure to aqueous environment at neutral pH. To accelerate the reactions, the samples were exposed to water vapor environment at 121 °C up to 48 h. XPS data were then collected with a photoelectron spectrometer (JPS-9010 MC; JEOL Ltd., Tokyo, Japan) equipped with an X-ray source of monochromatic MgK_α_ (10 kV, 10 mA). The vacuum in the chamber was set at 2 × 10^−7^ Pa, with analyzer pass energy at 10 eV and voltage step size at 0.1 eV. X-ray angle of incidence and takeoff angle were 34° and 90°, respectively. The XPS tests were validated at *n* = 6 for Si_3_N_4_ and Ti6Al4V alloy substrates, and at *n* = 8 for the Al_2_O_3_ substrate.

### 4.2. Cell Culture

KUSA-A1 mesenchymal bone marrow stromal stem cells (JCRB Cell Bank, Osaka, Japan) were cultured in a medium containing 4.5 g/L of glucose DMEM (d-glucose, l-glutamine, phenol red, and sodium pyruvate) supplemented with 10% fetal bovine serum. Cells were set on Petri dishes for 24 h at 37 °C.

### 4.3. Cell Proliferation and Metabolism

Disks of different materials were ultrasonically cleaned and UV-sterilized prior to being seeded with 1 × 10^5^/mL KUSA-A1 cells. They were then seeded with placing them into well plates in an osteogenic media (Dulbecco’s modified Eagle medium (DMEM) and 10 wt.% fetal bovine serum, ascorbic acid, hydrocortisone, and β-Glycerophosphate. This medium was refreshed twice weekly during a total of 14 days incubation period. Seeding of KUSA-A1 cells into empty well plates (made of silica glass) with and without the osteogenic medium served as positive and negative controls, respectively. The silica glass substrate was assumed to be completely inert toward cell metabolism and the cell response on it was only regulated by the presence or absence of osteogenic medium.

To monitor short-term proliferation, the cells were stained for fluorescence microscopy with Alexa Fluor^®^ 488 Phalloidin Thermo Fisher Scientific, Waltham, MA, USA) and Hoechst 33342 (Dojindo, Kumamoto, Japan) for 1 h. They were then washed three times with 1 mL TBST solution (mixture of Tris-Buffered Saline and Tween 20). Fluorescence spectroscopy images for cell counting were taken at 24 h with a fluorescence microscope of the type BZ-X700 (Keyence, Osaka, Japan). The number of cells per unit area was directly counted on fluorescence micrographs (total area of ~1 mm^2^ for each time and type of substrate). The results of cell counting were validated at *n* = 4 for each investigated substrate.

Some of the seeded discs for each type of material were also subjected to *in situ* laser Raman microscopy inspections (Raman-touch, Nanophoton, Osaka, Japan) to obtain information about the metabolism and the state of differentiation of the mesenchymal progenitor cells after very short-term (48 h) exposure to the respective substrates.

### 4.4. Bony Apatite Formation

After 14 days of culture on different substrates, a 3D laser-scanning microscope with a 150× objective lens at a numerical aperture of 0.9 (VK-X200, K series, Keyence, Osaka, Japan) was utilized to determine the volume of bony apatite formed on the biomaterial substrates. The volumetric amounts of bony tissue deposited by the cells per unit area was calculated from line scan profiles over the entire substrate using automatic software provided by the maker of the 3D laser-scanning microscope. The tests were validated at *n* = 6 for each investigated substrate. 

### 4.5. IGF-1 Measurement

IGF-1 signaling was used as a probe for measuring cell proliferation and differentiation efficiency. IGF-1 is a protein that stimulates both differentiation and apatite growth. It has been reported to be a main modulator for bone growth through endocrine/paracrine and autocrine mechanisms [11,74]. We employed the mouse IGF-1 ELISA (R&D Systems, Minneapolis, MN, USA) to measure IGF-1 concentration in the KUSA-A1 cell culture supernatants. Samples were first incubated for 1 h at room temperature, 0.1 mL of streptavidin solution was added, and the samples were incubated for 45 min at room temperature. Further addition of 0.1 mL of TMB one-step substrate reagent was made and, after incubation for 30 min at room temperature, 0.05 mL of stop solution was added. Optical densities at 450 nm were measured immediately after these procedures. The tests were validated at *n* = 4 for each investigated substrate.

### 4.6. Gla/Glu Osteocalcin Measurement

The Mouse Gla-Osteocalcin High Sensitive EIA Kit (Cat. #MK127; Takara Bio, Inc., Kusatsu, Shiga Prefecture, Japan) revealed Gla-type osteocalcin by means of a monoclonal antibody included in the used kit. Mouse Glu-Osteocalcin High Sensitive EIA Kit (Cat. #MK129; Takara Bio, Inc., Kusatsu, Shiga Prefecture, Japan) directly assessed decarboxylated osteocalcin from synthesized tissue by measuring enzymes peculiar to osteoclasts as well as the inactive (Glu-type) osteocalcin that they produce. Glu/Gla-osteocalcin EIA Kits could be directly used with cell-culture media containing fetal bovine serum because they did not cross-react with bovine antigens. These assays were applied according to manufacturer’s recommendations. Briefly, the Kit’s reagents (0.1 mL each) and the samples were made ready in separate well plates and then added to the Antibody Coated Microtiter plate at room temperature for 1 h. The reaction mixture was subsequently cast aside and the samples rinsed three times with 0.1% wash buffer (Tween 20/PBS). Successively, 0.1 mL of peroxidase (POD)-labeled Antibody Solution was poured in the wells with an 8-channel pipette and made reacting for 1 h at room temperature. The reaction mixture was set aside by washing four times with 0.1% wash Tween 20/PBS, 0.1 mL of Substrate Solution (TMBZ) at room temperature for 15 min. A highly viscous stop solution in a quantity of 0.1 mL was mixed into each well and kept for 1 h until formation of a stable color. Absorbance was then measured at 450 nm (zero value on distilled water) and plots constructed of concentration values of Gla/Glu-osteocalcin. Cells cultured on silica glass with and without the osteogenic medium were taken as positive and negative control samples, respectively. The experimental conditions adopted for these latter samples were the same as those applied to cells exposed to the tested substrates. Accordingly, we assumed that the silica glass substrate was completely inert toward cell metabolism and that, for the control silica glass substrate, the cell response was only regulated by presence or absence of osteogenic medium. The Gla/Glu-osteocalcin tests were validated at *n* = 4 for each investigated substrate.

### 4.7. ALP Stain Assay

The membrane-bound enzyme ALP, a marker of osteogenic differentiation, was measured on KUSA-A1 cells exposed to different substrates for nine days in osteogenic medium. The ALP activity was monitored upon staining with the TRAP/ALP Stain Kit (Wako, Osaka, Japan), according to the manufacturer’s instructions. ALP activity was assessed through direct pixel counting on optical micrographs.

### 4.8. Statistics

The elemental fractions of bonds detected by XPS on the surface of different surfaces, the number of cells per unit area, the concentrations of Glu/Gla-osteocalcin and IGF-1, and the volumetric amounts of bony tissue deposited by the cells per unit area were expressed as mean values ± one standard deviation. The results were statistically compared using one-way Analysis of Variance (ANOVA) on a sampling population, *n* = 4. A value of *p* < 0.003 was considered statistically significant and labeled with two asterisks.

## 5. Conclusions

The main conclusions of this paper can be summarized as follows:

(i) Si_3_N_4_ bioceramics presented osteoinductive properties by virtue of their peculiar surface chemistry, which evolved at the biomolecular interface with cell-friendly kinetics. The homolytic cleavage of Si-N bonds at the solid surface triggered a cascade of chemical reactions that could locally uplift pH and produce RNS (specifically NO) in low concentrations lying in the range of signaling used by cells in osteoblastogenesis crosstalk.
(ii) Neither Ti6Al4V alloy nor Al_2_O_3_ bioceramic substrates could be recognized to trigger osteoinductivity with respect to mesenchymal KUSA-A1 progenitor cells. Unlike the RNS involved with the Si_3_N_4_ surface chemistry, the ionic elution of metal species and the ROS involved with the surface chemistry of these two latter biomaterials were similarly detrimental to cell proliferation, their differentiation, and osteogenesis. Although not cytotoxic according to standard definitions of cytotoxicity, both these widely used biomaterials induced an apoptotic tendency in mesenchymal progenitor cells.
(iii) The present data partly answered a number of concerns raised by the statistics recently published by the National Consumer Affairs Center of Japan (and successively recognized by The Japanese Society of Oral Implantology) regarding malfunctioning of dental implants [94]. We believe that the origin of medium/long-term failures of dental implants is partly related to their adverse chemical interactions with the periprosthetic tissue in overlap with the severe environmental and mechanical stress conditions of the oral cavity.
(iv) The friendly exogenous NO kinetics of Si_3_N_4_ bioceramics could be further exploited in counteracting bone resorption pathologies and in promoting bone repair.
